# Extracellular Vesicles Mediate Communication between Endothelial and Vascular Smooth Muscle Cells

**DOI:** 10.3390/ijms23010331

**Published:** 2021-12-28

**Authors:** Marie Fontaine, Stéphanie Herkenne, Olivier Ek, Alicia Paquot, Amandine Boeckx, Cécile Paques, Olivier Nivelles, Marc Thiry, Ingrid Struman

**Affiliations:** 1 Laboratory of Molecular Angiogenesis, GIGA Research Center, University of Liège, B34, 1 Avenue de l’Hôpital, 4000 Liège, Belgium; marie.fontaine88@gmail.com (M.F.); stephanieherkenne@gmail.com (S.H.); olivierek0@gmail.com (O.E.); alicia.paquot@gmail.com (A.P.); a.boeckx@uliege.be (A.B.); cecilepaques@gmail.com (C.P.); olivier.nivelles@uliege.be (O.N.); 2 Laboratory of Cell and Tissues Biology, GIGA Research Center, University of Liège, 1 Avenue de l’Hôpital, 4000 Liège, Belgium; mthiry@uliege.be

**Keywords:** microRNA, extracellular vesicle, exosome, angiogenesis, miR-539, miR-582

## Abstract

The recruitment of pericytes and vascular smooth muscle cells (SMCs) that enwrap endothelial cells (ECs) is a crucial process for vascular maturation and stabilization. Communication between these two cell types is crucial during vascular development and in maintaining vessel homeostasis. Extracellular vesicles (EVs) have emerged as a new communication tool involving the exchange of microRNAs between cells. In the present study, we searched for microRNAs that could be transferred via EVs from ECs to SMCs and vice versa. Thanks to a microRNA profiling experiment, we found that two microRNAs are more exported in each cell type in coculture experiments: while miR-539 is more secreted by ECs, miR-582 is more present in EVs from SMCs. Functional assays revealed that both microRNAs can modulate both cell-type phenotypes. We further identified miR-539 and miR-582 targets, in agreement with their respective cell functions. The results obtained in vivo in the neovascularization model suggest that miR-539 and miR-582 might cooperate to trigger the process of blood vessel coverage by smooth muscle cells in a mature plexus. Taken together, these results are the first to highlight the role of miR-539 and miR-582 in angiogenesis and communication between ECs and SMCs.

## 1. Introduction

Blood vessels are initially formed during embryogenesis by the assembly of angioblasts, the endothelial cell (EC) precursors, which differentiate into a primary vascular plexus through a process called vasculogenesis [1]. Later, angiogenesis refines the network of vessels into arteries and veins. The recruitment and differentiation of vascular smooth muscle cells (SMCs) and pericytes are the main events during this progress. Whereas SMCs contribute to multiple concentric layers of arteries and veins, pericytes cover microvessels such as arterioles, capillaries, and venules.

The interaction between ECs and SMCs is fundamental to the formation and function of the vasculature [2]. These interactions not only occur during embryogenesis but are also involved in the dysfunction of the vascular homeostasis under some diseased conditions, including atherosclerosis and cancer [3,4]. These EC–SMC dialogues were proven to occur in several ways, including direct interactions and exchange of growth factors. In this work, we would like to determine whether EC and SMC also communicate via the exchange of extracellular vesicles (EVs).

Many studies have documented the biological role of EVs as mediators of cell communication [5]. EVs are secreted by most, if not all, cell types. The content of EVs varies and can include small non-coding RNAs, mRNAs, proteins, and lipids. EVs have been classified according to their sub-cellular origin [6,7]. EVs that are directly formed and released from the cellular plasma membrane are called microparticles, microvesicles, or ectosomes and display a diverse range of sizes (100–1000 nm in diameter). Internal vesicles generated within multivesicular endosomal compartments are secreted in the extracellular milieu when these compartments fuse with the plasma membrane: these EVs are termed exosomes. Exosomes are classically defined by their size (50–100 nm) and their content in endosome-associated proteins [8]. For the purpose of this work, we will use the general term “EVs” since the subcellular origin of the vesicles was not determined.

Due to their localization in biofluids, EVs have emerged as powerful biomarkers for several diseases, including cancer [9]. Functionally, EVs are now considered mediators of cell–cell communication [5,10]. Most studies identified microRNAs that are secreted by donor cells and transferred to recipient cells in which microRNAs can exert their functions.

Several studies reported that EVs secreted from an endothelial cell origin are important players in endothelial function and contribute to angiogenesis-related disease such as cardiovascular disease or cancer. Numerous cancer studies have highlighted the pro-angiogenic properties of EVs secreted by tumor cells from various cancer types (for a review, see [11]). Within the tumoral environment, the exchange of materials via EVs has been observed from tumor cells to endothelial cells, as well as in the reverse direction [12]. For example, we previously showed that EC sheds microRNAs via EVs that can prevent tumor growth [13]. However, ECs can also receive EVs from their environment that modulate their phenotype. For instance, mesenchymal stem cells (MSCs) were found to secrete EVs that can promote angiogenesis in vitro and in vivo [14]. EVs from endothelial cells can also autonomously modulate their own behavior. For example, endothelial cells secrete miR-214–enriched EVs that stimulate the migration and angiogenesis of a neighboring EC-recipient cell, allowing for the stimulation of blood vessel formation [15]. EVs secreted by SMCs have also been implicated in several processes, such as SMC calcification in atherosclerotic plaques and vascular coagulation [16].

To date, a few publications reported a transfer of microRNAs between ECs and their associated SMCs [17,18,19]. EVs shed from ECs were shown to contribute to the SMC contractile phenotype [20]. However, EVs released by SMCs are transferred to ECs to promote arteriosclerosis [21]. In the present study, we aim to identify new actors included in EVs that regulate angiogenesis. Using a coculture model, we identified two microRNAs, miR-539 and miR-582, which could be involved in the communication between two cell types and the modulation of ECs and SMCs functions. Importantly, the microRNAs encapsulated in EVs can also act autonomously to modulate their own phenotypes.

## 2. Results

### 2.1. ECs and SMCs Are Able to Communicate via EVs

To investigate the extracellular communication occurring between ECs and their associated SMCs, we investigated whether cells could incorporate EVs from the other cell type. We isolated EVs produced by ECs (EC EVs) and HUVSMCs (SMC EVs) using ultracentrifugation. We first characterized EVs for the presence of endosomal markers. Using Western blot analysis, we confirmed the presence of the CD63 marker in EVs lysates as well as CD81 and CD9, two other exosomal markers [8,22] (Supplementary Figure S1a). Using dynamic light scattering, we analyzed the size of the purified vesicles and observed that EC EVs and SMC EVs show a typical size range, with a maximum peak at 90 and 80 nm, respectively (Supplementary Figure S1b). We further characterized EVs by transmission electron microscopy, confirming the presence of CD63 and CD81 exosomal markers (Supplementary Figure S1c) and size. The presence of CD63 at the EV surface was confirmed by flow cytometry analysis in EC and SMC EVs (Supplementary Figure S1d).

**Figure 1:**
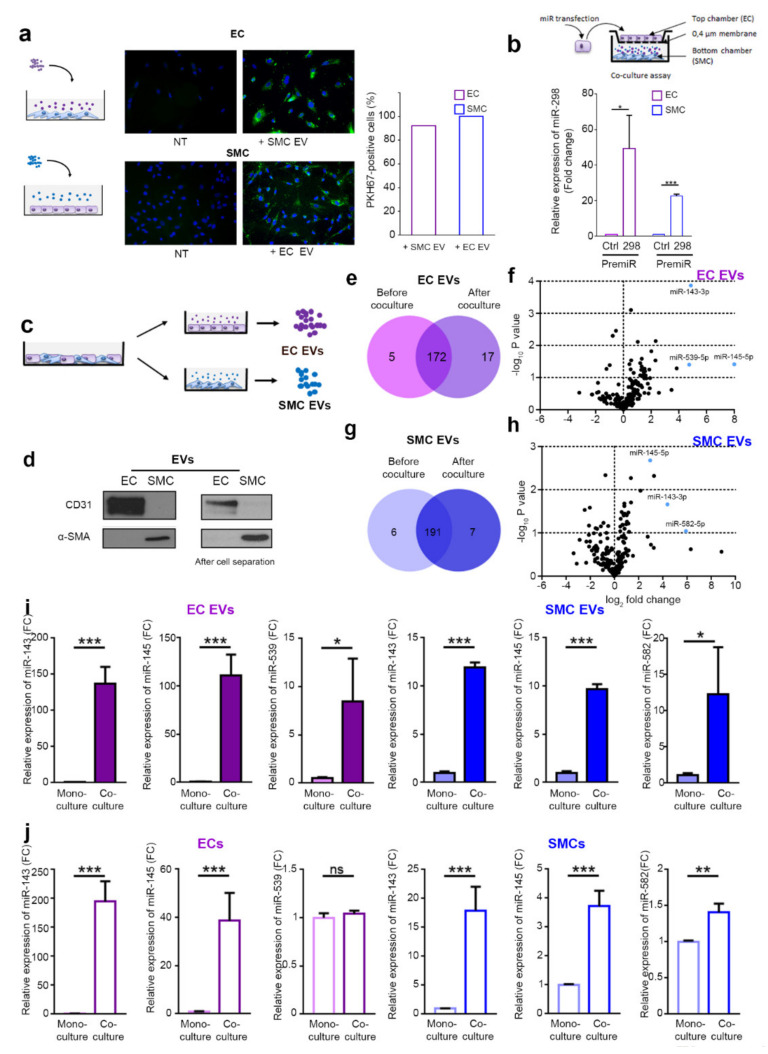
ECs and SMCs communicate via EVs. (**a**) Fluorescence microscopy detection of the uptake of PHK67-labeled EVs (green) by ECs and SMCs (DAPI, blue), scale bars = 50 µm. Graph shows the percentage of positive cells for PKH67. (**b**) Overview of the experimental setup: miR-298 levels evaluated qPCR in SMCs cultured with ECs transfected with pre-miR-control or pre-miR-298 (blue bars). The purple bars show the reverse experiment in which the level of miR-298 was analyzed in EC cultured in presence of SMC transfected with pre-miR control or pre-miR-298. (**c**) Overview of the experimental setup. ECs and SMCs were cocultured for 48 h, separated using magnetic beads, and seeded as monoculture to produce EVs. microRNA content of cells and EVs were analyzed by microRNA qPCR profiling. (**d**) Western blot of CD31 and α-SMA in EVs isolated from EC and SMC cultures (left panels) or after coculture and separation of cells with CD31-coated magnetic bead (right panels) (**e**) Diagram of miRNAs common and specific to EC EVs before and after coculture. (**f**) Volcano plot of fold changes (log_2_ values) and probability values (−log_10_) for individual miRNAs in the EVs before and after coculture. (**g**) Diagram of miRNAs common and specific to SMC EVs before and after coculture. (**h**) Volcano plot of fold changes (log_2_ values) and probability values (−log_10_) for individual miRNAs in the SMC EVs before and after coculture. (**i**) Analyses of the expression of miR-143, miR-145, and miR-539 by qPCR in EC EVs before and after coculture with SMCs and the expression of miR-143, miR-145, and miR-582 in SMC EVs before and after coculture with ECs. (**j**) Analyses of the expression of miR-143, miR-145, and miR-539 by qPCR in ECs before and after coculture with SMCs and the expression of miR-143, miR-145, and miR-582 in SMCs before and after coculture with ECs. All data are the mean ± SEM (N = 3 independent experiments). * *p* < 0.05, ** *p* < 0.01, and *** *p* < 0.001 vs. the respective control.

Next, to determine whether cells could incorporate EVs from the other cell type, we labeled the EVs produced by ECs and SMCs with the fluorescent green lipid dye PKH67 and incubated the labeled EVs with the SMCs and ECs, respectively (Figure 1a). An analysis of the EVs uptake, performed by fluorescence microscopy, revealed that EVs from both cell types can be incorporated in the other cell type (Figure 1a). Then, we examined whether microRNAs could be transferred between ECs and SMCs via EVs. We decided to follow the transfer of an exogenous mouse microRNA that is not conserved in human cells, mmu-miR-298. This was achieved by transfecting the cells with the pre-miR-298. After verifying the transfection efficiency by qPCR (Supplementary Figure S1e,f), transfected ECs were cocultivated with non-transfected SMCs in a transwell coculture system, where the two cell types were separated by a membrane with 0.4 µm pores. Using qPCR, we observed a transfer of this microRNA in the SMCs (Figure 1b). In the reverse experiment, SMCs could also incorporate mmu-miR-298 from EVs released by ECs. The lower level of incorporation observed in SMCs is probably due to a reduced transfection efficiency of ECs compared to SMCs (Supplementary Figure S1e,f). From those experiments, we can conclude that ECs and SMCs could exchange microRNAs via EVs.

### 2.2. The microRNAs Cargo of EVs Is Modified by the Coculture

We then hypothesize that EC–SMC communication could occur via the exchange of microRNA via EVs. To test this hypothesis, we set up coculture experiments in which ECs and SMCs were cocultured, then separated using CD-31 coated magnetic beads, and finally seeded as a monoculture to produce EVs (Figure 1c). This method enabled us to obtain cell-type-specific EV samples after the coculture step, as assessed by Western blot analysis for CD31, an endothelial marker, and α-smooth muscle actin (α-SMA), a SMC marker, that are specifically present in ECs and SMCs EVs, respectively (Figure 1d). To analyze the microRNAs content of the EVs, we performed a qPCR-based microRNA profiling of EVs produced by ECs alone, SMCs alone, or cells that were in coculture. This qPCR-based profiling showed that the majority of microRNAs were not regulated after the coculture of ECs and SMCs. However, we observed some microRNAs that were only present in EVs after the coculture (Figure 1e–h and Supplementary Table S1), which, according to our hypothesis, could be implicated in the intercellular communication between ECs and SMCs. Among these miRNAs, miR-143-3p (miR-143), miR-145-5p (miR-145), and miR-539-5p (miR-539) were highly present in ECs EVs from coculture compared to ECs EVs before coculture (Figure 1f). Similarly, miR-143, miR-145, and miR-582-5p (miR-582) were more present in SMC EVs isolated from the coculture compared to the SMSs EVs before coculture (Figure 1f,h). Of note, miR-582 is not regulated in ECs EVs by the coculture system, nor is miR-539 in SMCs EVs (Supplementary Table S1). These regulations were confirmed by TaqMan microRNA assays, another qPCR technique (Figure 1i). To investigate whether the change in microRNA level inside the EVs is caused by a cellular adaptation during the coculture, we measured their cellular level before and after the coculture. Interestingly, while miR-143 and miR-145 are strongly upregulated in both cell types after coculture, miR-539 in ECs and miR-582 in SMCs are, respectively, not or weakly regulated by the coculture conditions (Figure 1j). Furthermore, those microRNAs are likely to be encapsulated inside the vesicles since RNAse treatment does not significantly reduce their levels in contrast to the treatment of EV preparations with detergent to lyse membrane vesicles, in combination with RNase digestion (Supplementary Figure S1g,h). Since our aim was to identify the microRNAs that could specifically be exchanged by EVs in coculture condition, we decided to focus on miR-539 ad miR-582, the only ones regulated in only one cell type.

### 2.3. miR-539 and miR-582 Modulate ECs and SMCs Functions In Vitro

When EVs are secreted in the extracellular milieu, they can act on neighboring cells of the same type, as well as on cells from another cell type. For example, in a coculture system, EVs released from ECs could modulate SMCs’ behavior and ECs’ phenotype. We decided to study the impact of both microRNAs on both cell types. Therefore, we performed gain- and loss-of-functions studies by transfecting cells with pre-microRNAs (pre-miRs) or anti-microRNAs (anti-miRs), respectively. Transfection efficiency was verified by qPCR (Supplementary Figure S2a–d).

**Figure 2:**
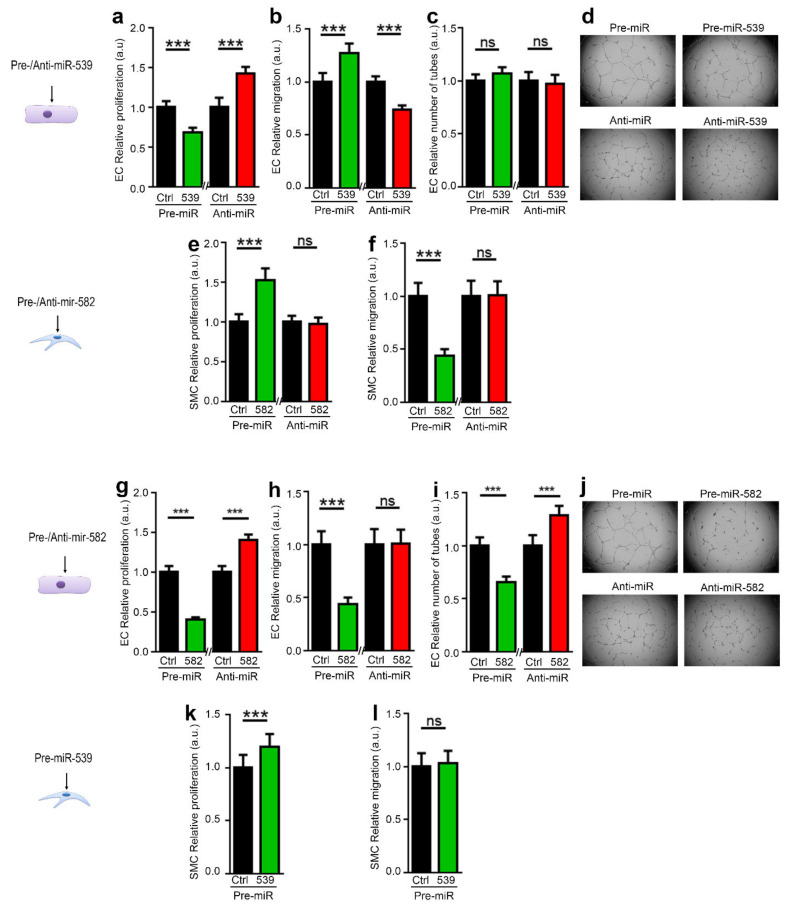
miR-539 and miR-582 modulate EC and SMC functions in vitro. ECs were transfected with pre-miR-539 or a pre-miR-Control or with an anti-miR-control or an anti-miR-539 (**a**–**d**). SMCs (**e**,**f**) or ECs (**g**–**j**) were transfected with pre-miR-582 or a pre-miR-control or with an anti-miR-control or an anti-miR-582. (**k**,**l**) SMCs were transfected with pre-miR-539 or a pre-miR-control. Cells were then used in the following bioassays: (**a**,**e**,**g**,**k**) proliferation assay, (**b**,**h**) migration wound-healing assay of ECs, (**l**,**f**) migration of SMCs in boyden chamber assay, and (**c**,**d**,**i**,**j**) tubulogenesis assay of ECs. Right: representative images from d and j are shown. All data are the mean ± SD (*n* = 3 independent experiments). *** *p* < 0.001 vs. the respective control.

We first determine whether the miRs could act on the cell of origin. As shown in Figure 2a–d, the transfection of miR-539 in EC decreases its proliferation and increases its migration (Figure 2a,b). Interestingly, while miR-539 does not affect the ability of endothelial cells to form vessel-like structures (Figure 2c,d), in SMCs, increasing the level of miR-582 increases their proliferation and reduces their migration (Figure 2e,f).

Then, we analyzed whether the miRs could act on the other cell type. The transfection of EC with miR-582, which we found to be upregulated in SMC, could regulate EC behavior by decreasing their proliferation. While no impact was observed on EC migration (Figure 2h), miR-582 reduces the ability of endothelial cells to form vessel-like structures (Figure 2j). miR-539 could also impact smooth muscle cells’ behavior since miR-539 transfection in SMC increases their proliferation (Figure 2k), but we did not observe any impact on the migration of SMCs after the modulation of miR-539 levels (Figure 2i). Interestingly, blocking the endogenous miR-539 or miR-582 in EC using anti-miR transfection shows the opposite effects. Unfortunately, in SMCs, we could not assess the effect of anti-miR-539, since its transfection in not efficient in those cells (Supplementary Figure S2d).

### 2.4. miR-539 and miR-582 Can Be Transferred via EVs and Modulate ECs Functions In Vitro

**Figure 3:**
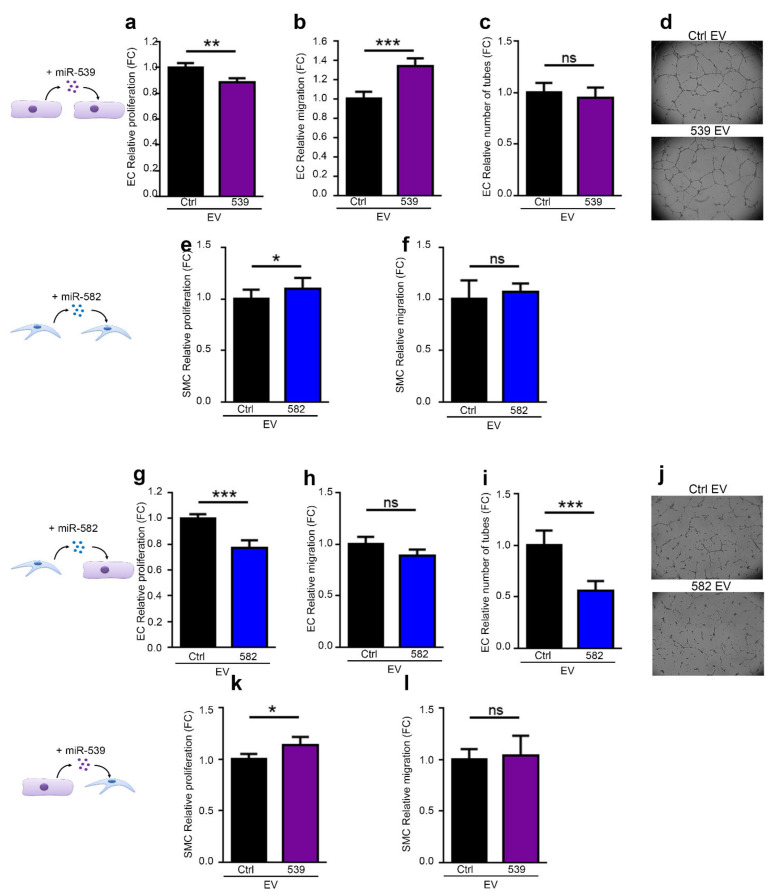
miR-539- and miR-582 can be transferred via EVs and modulate EC and SMC functions in vitro. EVs were purified from ECs transfected with pre-miR-539 (539 EV) or a pre-miR-Control (Ctrl EV) (**a**,**b**,**k**,**l**), or from SMCs transfected with pre-miR-582 (582 EV) on a pre-miR-control (Ctrl EVs) (**e**–**j**), and used in the following bioassays, (**a**,**g**) EC proliferation, (**b**,**h**) migration wound-healing assay of ECs, and (**c**,**d**,**i**,**j**) tubulogenesis of ECs. Right: representative images from (**c**) and (**i**) are shown, (**e**,**k**) proliferation of SMCs, and (**f**,**l**) migration of SMCs in Boyden chamber assays. All data are the mean ± SD (*n* = 3 independent experiments). * *p* < 0.05, ** *p* < 0.01 and *** *p* < 0.001 vs. the respective control.

To determine whether the effects observed above could be assigned to EV delivery, we produced EVs with an elevated content of both miRNAs. To do so, we transfected ECs and SMCs with miR-539 and miR-582, respectively, and purified their EVs. qPCR results showed that miR-539 and miR-582 transfection drastically increased the level of both microRNAs in EVs (Supplementary Figure S3). Although less pronounced, most of the effects observed with the transfected cells were confirmed with EVs enriched with miR-539 and miR-582. As expected, ECs treated with miR-539-loaded EVs showed a decrease in proliferation and an increase in migration, while no impact was observed in the tubulogenesis assay (Figure 3a–d). However, the effects of miR-582-loaded EVs on SMCs are very weak in terms of proliferation and not observed for migration (Figure 3e–f). Conversely, the effect of miR-582 encapsulated in SMC EVs showed a decreased proliferation and ability to form a tubular network on matrigel in ECs (Figure 3g,j). In the other cell type, SMC, the inhibition of proliferation and absence of migratory properties were confirmed with miR-539-loaded EVs (Figure 3k,l). The results confirm the effects observed with the transfection of the cells, even though the effects are less pronounced with EVs.

### 2.5. miR-539 and miR-582 Target Many Genes in ECs and SMCs

**Figure 4:**
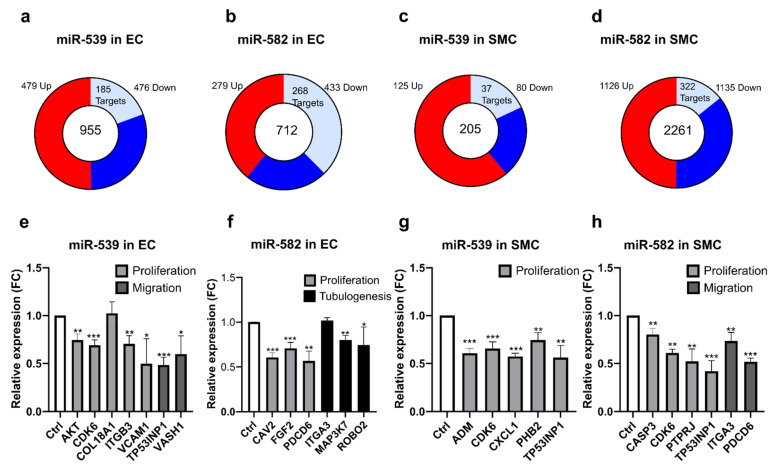
miR-539 and miR-582 regulate genes involved in angiogenesis-related biological processes in ECs and SMCs. (**a**,**d**) Transcriptomic analysis of ECs transfected with pre-miR-539 (**a**) and pre-miR-582 (**b**) and SMCs transfected with pre-miR-539 (**c**) and pre-miR-582 (**d**). Pie chart shows all significant regulated genes (*p* < 0.05) analyzed by RNA sequencing with the number of upregulated genes colored in red while downregulated genes are labeled in blue. Light blue shows number of potential microRNA targets. (**e**–**h**) Validation of some putative targets by qPCR. (**e**) ECs were transfected with pre-miR-control or pre-miR-539. (**f**) ECs were transfected with pre-miR-control or pre-miR-582. (**g**) SMCs were transfected with pre-miR-control or pre-miR-539. (**h**) SMCs were transfected with pre-miR-control or pre-miR-582. All data are the mean ± SD (*n* = 3 independent experiments). * *p* < 0.05, ** *p* < 0.01, and *** *p* < 0.001 vs. the respective control.

To obtain an overview of the genes modulated by miR-539 and miR-582, we performed an RNA sequencing on total RNA from ECs and SMCs transfected with microRNA mimics. A list was generated of genes that are differentially expressed, comparing control transfection and miR-539 or miR-582 (see Figure 4a–d and Supplementary Table S2). We analyzed this list with the Gene Ontology database (http://geneontology.org/, accessed on 23 February 2016) to highlight the biological processes by which those microRNAs could be implicated. Consistent with our experiments, GO terms associated with migration, proliferation, tube development, and more general terms such as angiogenesis and vascular development were highlighted by gene ontology analysis (Table 1). To further identify the gene targets that could be regulated by the microRNAs, we searched into these lists for some potential targets generated by the algorithm TargetScan (http://www.targetscan.org, accessed on 3 February 2016). We observed that many genes were regulated by miR-539 and miR-582 in our cells and could explain our results. Heatmaps depict the regulation of each microRNA in both cell types and indicate the potential targets (Supplementary Figures S4 and S5). We then confirmed some of the genes selected in each GO network by qPCR. We were able to confirm the miR-539 regulations in ECs: AKT1, CDK6, ITGB3, CAM and TP53INP1, and VASH1, which are, respectively, part of the migration and proliferation GO networks. (Figure 4e). In miR-582-overexpressing ECs, we observed a decrease in the expression of CAV2, FGF2, and PDCD6 (GO:proliferation) and MAP3K7 and ROBO2 (GO: tubulogenesis) (Figure 4f). In SMCs, miR-539 regulated the expression of ADM, CDK6, CXCL1, PHB2, and TP53INP1 (GO: proliferation, Figure 4g). Finally, in SMCs, miR-582 decreased the expression of CASP3, CDK6, ITGA3, PTPRJ, TP53INP1 (GO: proliferation), and PDCD6 (GO: migration) (Figure 4h).

### 2.6. miR-539 and miR-582 Participate in Blood Vessel Maturation

Since miR-539 and miR-582 are only detected in EVs when ECs and SMCs are cultured together, we wondered whether these microRNAs could impact the blood vessel development in a physiological model of neovascularization, in which both cell types orchestrate the vasculature establishment. The mouse retinal vasculature is an excellent model to study vascular growth and remodeling during development [23]. Therefore, we decided to inject EVs loaded with the microRNA, i.e., EC EVs loaded with miR-539 and SMC EVs loaded with miR-582 in newborn pups, and observed the vascularization of the retina.

**Figure 5:**
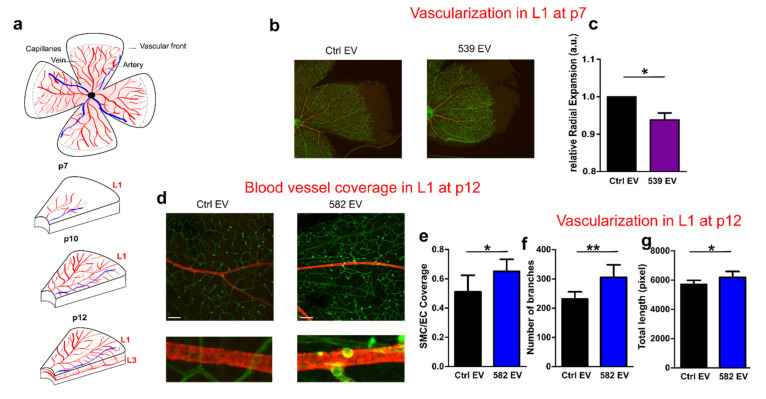
miR-539 EVs and miR-582 EVs participate in vascular remodeling of the retina. (**a**) Cartoon illustrating the development of the superficial (L1), intermediate (L2), and deep vascular plexus (L3). (**b**) Left: retinal flat mount representation. For (**b**–**g**), EVs were purified from ECs transfected with pre-miR-539 (539 EV) or a pre-miR-Control (Ctrl EV) or from SMCs transfected with pre-miR-582 (582 EV) on a pre-miR-control (Ctrl EV) (**b**) Isolectin-B4 staining on Postnatal Day 7 retinas from pups that were injected at Postnatal Day 1 with EVs. Scale bar: 25 µm. (**f**–**h**). The histograms represent the quantification of the radial expansion from the optic nerve to the vascular front (**c**), N = 8 eyes, two independent experiments. (**d**) Confocal images of isolectin-B4/α -SMA staining on postnatal Day 12 retinas from pups that were injected at Postnatal Day 7 with miR-582 EVs in the superficial layer (L1). Scale bar: 200 µm. (**e**–**g**). The histograms represent the quantification of the coverage of ECs by SMCs (Spearman’s rank correlation value) (**e**), the number of branches (**f**), and the total length of isolectin+ vessels (**g**) * *p* < 0.05, ** *p* = 0.01.

Mouse retina is avascular at birth, and blood vessels progressively sprout from the center towards the periphery until Postnatal Day 7 (P7) (Figure 5a). From P5, arterioveneous differentiation and pericytes/SMCs coverage start to occur. From P7, blood vessels from the superficial (L1) vascular plexus start to sprout inside the retina to form the deeper plexus (L3). Then, a tertiary deeper layer grows between the L1 and L3 at Day 12. We first decided to assess vascular growth at an early stage. Injection intra-peritoneously from P1 up to P3 with miR-539 EVs reduces the radial expansion of the vascular network of pups at P5 compared to control EVs (Figure 5b,c). Nevertheless, miR-582 EVs do not affect vessel growth at that stage (data not shown). To assess the impact of miR-582 EVs on SMCs, we decided to intra-peritoneously inject the pups with miR-582 EVs from P6 up to P8. miR-582 EVs slightly increase the branching and length of the vascular network and also increases the endothelial coverage by smooth muscle cells revealed by α-SMA labeling (Figure 5d–g). miR-539 EVs do not show any phenotype at that stage (data not shown). Furthermore, to assess vascular growth at a later stage, retinas were observed at Day 12, when a new vascular sprouted from the L1, invaded the retina and established L3. In agreement with the absence of SMCs in this layer at that stage, miR-582 EVs do not affect this neovascularization in this newly forming vascular layer (Supplementary Figure S5a–c). These results indicated that miR-539 and miR-582 might together trigger blood vessel coverage by smooth muscle cells in a mature plexus.

## 3. Discussion

In recent years, EVs have emerged as new players in intercellular communication [5]. It has been shown in several studies that EVs can contain genetic information that could be transferred from cell to cell [24,25,26], but only a few publications have presented that transfer between ECs and their associated SMCs [18,19]. In this study, we wanted to identify microRNA that could function in the cross talk between these two cell types. Furthermore, we also aimed to study if those microRNAs could also affect the behavior of their cell of origin.

**Figure 6:**
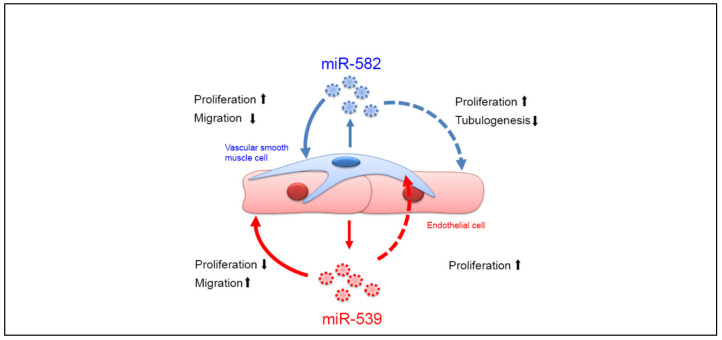
ECs and SMCs communicate via the exchange of microRNAs-loaded EVs. The coculture of ECs and SMCs induces a modification to the microRNAs’ cargo of EVs. After the coculture, ECs EVs are enriched in miR-539 while SMCs EVs are enriched in miR-582; then, cells exchange their EVs, inducing some modification to their cellular functions.

In this study, we showed that coculture modifies the miRNAs content of the EVs and we highlighted two new miRNAs that play a role in communication between ECs and SMCs. Profiling experiments allowed us to identify an increase in miR-143 and miR-145 levels in the cell, as well in their EVs. As demonstrated before [18,19], these miRNAs are involved in communication between ECs and SMCs. Interestingly, we found that the levels of two other microRNAs, miR-539 and miR-582, were elevated in EVs, while their cellular levels were unchanged after the coculture. These miRNAs have already been proven to regulate cell proliferation and cell migration in several cancers [27,28]. Both microRNAs seem to act as tumor-suppressor genes. We then investigated the functions of these miRNAs in ECs and in SMCs by performing experiments with miRNA expression modulators and miRNA-loaded EVs. The effects of miR-539 and miR-582 on the functions of cells are summarized in Figure 6. As ECs and SMCs exchange their miRNA-loaded EVs, ECs show a decrease in proliferation and the ability to form tubes, and SMCs show an increased proliferation. During angiogenesis, the maturation of blood vessels is characterized by the recruitment of SMCs and the decrease in ECs functions [4]. miR-539 and miR-582 could, therefore, be implicated in this phase of the process. Indeed, thanks to the retinal model, we observed that miR-582 increases the coverage of ECs by SMCs. Since this microRNA does not show any effect on the growing vasculature, either at P7 or in the newly formed vessels in the deep layer at P12, this microRNA might act only on mature vasculature. This observation is in agreement with the fact that miR-582 is secreted by the SMCs that are present at Day 7. In this model, the impact of miR-539 is less obvious, since no effect was observed either on ECs or SMCs when both cell types were present (i.e., after P7). Only an autonomous role was observed for ECs before the colonization of blood vessels by SMCs. Nevertheless, we could expect that miR-539 would act at the latter stage in the steps, when the initially dense vascular network of the SP undergoes remodeling processes after P10 until adulthood [23]. In addition, we also observed an autonomous regulation of ECs and SMCs (Figure 6). The incorporation of miR-539-loaded EVs by ECs decreases the proliferation of the cells and increases their migration. On the other hand, the incorporation of miR-582-loaded EVs by SMCs stimulates their proliferation but decreases their migration. It seems that these cells are able to modulate their own functions, reinforcing the effects observed during previous experiments. This phenomenon has already been observed in cancer cells, which can stimulate their own migration and invasion through the incorporation of their extracellular vesicles [29].

Numerous reports have shown that cells secrete a heterogeneous population of EVs with different sizes and composition [30,31]. For example, EVs positive for CD63, a widely used exosome marker, represent only a fraction of EVs purified from dendritic or Hela cells [32]. Therefore, while we observed by Western blot analysis that the method of cell isolation led to cell-type specific EVs, it is likely that the pool of EC EVs and SMC EVs found positive for, respectively, CD31 and SMC markers probably represents a subpopulation of all EVs present in the preparation. In addition, the isolation by ultracentrifugation causes non-vesicular macromolecule contamination that could bind to microRNAs like RNA-binding proteins [33]. Therefore, when analyzing RNA content in EVs, it is important to perform an RNAse A treatment before RNA extraction that will degrade non-vesicular RNA. Since this treatment does not significantly reduce the level of miR-539 and miR-582 in EVs in this work, it suggests that those microRNAs are encapsulated into vesicles and protected from degradation. Nevertheless, since we did not use proteinase K treatment together with RNAse, we cannot completely rule out that some miRNAs isolated in the EV preparations are associated with high molecular weight protein complexes as mentioned in [34].

Finally, we performed an RNA-sequencing experiment to identify potential targets of miR-539 and miR-582. We confirmed several genes that were downregulated following the overexpression of miR-539 or miR-582, which could explain the results obtained in the functional assays. We observed in ECs that miR-539 regulate the expression of AKT1, CDK6, ITGB3, and VCAM1, known to be implicated in cell proliferation, and the expression of TP53INP1 and VASH1 that is implicated in cell migration [35,36]. Interestingly, it was shown that AKT1 induces the phosphorylation of ITGB3 to induce cell proliferation [37]. As AKT1 and ITGB3 are both modulated by miR-539, these results show that a single miRNA can target several genes implicated in a biological process, increasing the incidence of the miRNA on the considered processes. We observed that miR-582 decreases the expression of CAV2, FGF2, and PDCD6, implicated in the ECs’ proliferation [38,39,40]. Moreover, miR-582 also decreases the expression of MAP3K7 and ROBO2, which is known to play a role in the tubulogenesis of ECs [41,42,43]. FGF2 is also implicated in the tube-formation ability of ECs [44]. In SMCs, we observed that miR-539 and miR-582 both regulate the expression of CDK6 and TP53INP1. It is interesting to note that CDK6 has the opposite effect on the proliferation of ECs and SMCs. This phenomenon can be explained by the expression of p16^INK4a^, which is different in ECs and SMCs (fold ≅20, data not shown). Less expressed in ECs, p16^INK4a^ is not able to inactivate CDK4 and CDK6 to stop the cell cycle. However, p16^INK4a^ is overexpressed in SMCs and is able to interact with CDK4 and CDK6 to block the cell cycle [45]. Our results show that the same protein can have different effects on proliferation, depending on the cell type and expression of their cofactors. miR-539 also decreases the expression of ADM, CXCL1, and PHB2, implicated in the SMCs proliferation [46,47,48]. Moreover, PHB2 has already been confirmed as a target gene of miR-539 [49]. The proliferation of SMCs is also under the influence of miR-582, as this miRNA decreases the expression of CASP3 and PTPRJ [50,51]. Finally, we confirmed the regulation of ITGA3 and PDCD6 by miR-582, which are implicated in cell migration [52,53].

To the best of our knowledge, these results report, for the first time, miR-539 and miR-582′s possible involvement in vesicular communication between ECs and their associated SMCs. Our data reveal that a coculture of both cell types influences the miRNA cargo of their EVs. In this context, miR-539 and miR-582 could be involved in the maturation process of blood vessels since we observed a reduction in ECs functions when the SMCs are stimulated. Although several studies have shown that the microRNAs can autonomously regulate the cell of origin, i.e., miR-539 on EC [54,55] and miR-582 on SMC [56], it was not shown that both miRNAs can act on the other cell type. In light of our results, we suggest that the rupture of correct communication via EVs between ECs and SMCs could lead to a disequilibrium, in which miR-582 secreted by SMCs might impact ECs function. The use of therapeutic EVs is a new approach to specifically deliver microRNAs and might be considered a novel potential therapeutic approach for thrombotic or other vascular disorders.

## 4. Material and Methods

### 4.1. Cell Culture

The isolation of Human Umbilical Vein Endothelial Cells (HUVECs, here called ECs) was previously described [57]. ECs were cultured on gelatin-coated flasks in in heparin-free EGM2 (Lonza, Verviers, Belgium) supplemented with full 5% FCS (Gibco, Thermo Fisher Scientific, Waltham, MA, USA) (*v*/*v*) or EV-depleted serum. To prepare EV-depleted serum, heat-inactivated FCS was ultracentrifuged overnight at 110,000× *g* at 4 °C and the pellet was discarded. Human umbilical vein smooth muscle cells (HUVSMCs, here called SMC (ScienCell, Carlsbad, CA, USA) were cultured in flasks coated with poly-L-lysine (2 µg/cm^2^) in SMCM medium (ScienCell). Cells were used between Passages 3 and 8.

### 4.2. EVs Isolation

EVs were isolated and purified from the supernatants of cell cultures using differential centrifugations. Cells were cultured in EGM-2 medium supplemented with EVs-depleted serum. After 72 h, medium was collected and centrifuged at 2000× *g* for 20 min at 4 °C, then at 12,000× *g* for 45 min at 4 °C. Supernatants were filtered through a 0.22 µm filter (Millipore) and ultracentrifuged at 110,000× *g* for 2 h at 4 °C (SW32 TI rotor, Beckman Coulter). The pellets were washed with phosphate buffer saline (PBS) and ultracentrifuged again at 110,000× *g* for 2 h at 4 °C. The pellets were re-suspended in PBS. Protein levels of EVs preparations were measured using the BCA Protein Assay kit (Pierce) following the manufacturer’s instructions.

### 4.3. PHK67 Labelling of EVs

EVs were labeled with a PKH67 Green Fluorescent cell linker kit for cell membrane labeling (Sigma) according to the manufacturer’s instructions. In brief, EVs were isolated as described above. After the first ultracentrifugation, the EV pellet was resuspended in 1 mL of diluent C with 2 µL of PKH67. After a 5 min incubation at room temperature, neutralization was performed by adding 1 mL PBS/FBS 20%. Labeled EVs were washed by performing two ultracentrifugation cycles (110,000× *g*, 2 h, 4 °C): resuspension in PBS EC and SMS were seeded on coverslip coated with gelatin 0.2% for 45 min at 37 °C (30,000 cells in a 24-well plate). A total of 5 µg of PKH67 labeled EVs were incubated for 6 h. Cells were fixed with PFA (1%) and DAPI 0.1% washed twice with PBS and mounted on a slide for observation under a fluorescent microscope.

### 4.4. Western Blot Analysis

Cells were washed with PBS and scraped into a RIPA lysis buffer (50 mM Tris (pH = 7.5), 150 mM NaCl, 10 mM CaCl_2_, 0.5% NP-40, 0.25% sodium desoxycholate, 0.1% SDS, protease inhibitor cocktail cOmplete Mini EDTA free (Roche), and 0.2% octylglucoside). Cell debris was removed by centrifugation at 10,000× *g* for 15 min. EVs were lysed using a 1% Triton X-100/0.1% SDS buffer. Protein concentrations were measured using the BCA Protein Assay kit (Pierce), following the manufacturer’s instructions. A total of 20 µg of protein-lysates were resolved using SDS-PAGE and transferred to polyvinlidene fluoride membranes (Millipore). Blots were blocked for 1 h with 5% milk in Tris-buffered saline with 0.1% Tween 20 (TBST). Then, blots were probed overnight at 4 °C with the following primary antibodies: anti-CD63 (556019, BD Biosciences, Erembodegem, Belgium), anti-CD9 (sc-20048, Santa Cruz Biotechnology), anti-CD81 (10630D, Invitrogen), anti-CD31 (M0823, Dako), and anti-α-SMA (M0851, Dako). After three washes with TBST, blots were incubated for 1 h with a peroxidase-conjugated secondary antibody (7076, Cell Signaling, Bioké, Leiden, The Netherlands). The detection was performed using a chemilunescent system (ECL; Pierce, Thermo Fisher Scientific, Merelbeke, Belgium).

### 4.5. Dynamic Light Scattering

EVs were suspended in PBS at a concentration of 50 µg/mL and analyzed in a Zetasizer Nano ZS (Mavern Instruments, Palaiseau, France). Intensity, volume, and distribution data for each sample were collected on a continuous basis for 20 runs of 10 s. Protein concentrations were measured using the BCA Protein Assay kit (Pierce), following the manufacturer’s instructions.

### 4.6. Electron Microscopy of Whole-Mounted Immuno-Labelled EVs

EVs were placed on Formvar-carbon coated nickel grids for 1 h, washed three times with PBS, and fixed with 2% paraformaldehyde for 10 min. After three washes, grids were then incubated for 2 h with the following antibodies: anti-CD63 (10628D, Invitrogen, Thermo Fisher Scientific) and anti-CD81 (10630D, Invitrogen). EVs were then washed five times and incubated with a 10 nm-gold labeled secondary antibody. They were washed five more times and post-fixed with 2.5% glutaraldehyde for 10 min. Samples were contrasted using 2.5% uranyl acetate for 10 min, followed by four washes and an incubation of 10 min in lead citrate. Grids were finally washed four times in deionized water and examined with a JEOL JEM-1400 transmission electron microscope at 80 kV.

### 4.7. Flow Cytometry

EVs were incubated for 30 min with latex beads (Invitrogen). The EVs-coated beads were saturated for 30 min in PBS containing 5% BSA, then incubated for 1 h at 4 °C with an anti-CD63 antibody (556019; BD Biosciences). The beads were incubated with a biotin-coupled secondary antibody (E0433, Dako, Agilent Technologies Belgium, Heverlee, Belgium) for 30 min, incubated with streptavidin-PE (016-110-084, Jackson ImmunoResearch, Ely, UK) for 30 min, and analyzed on the FACSCalibur flow cytometer (BD Biosciences).

### 4.8. Cells Transfections and Treatments

For functional assays performed with cells transfected with pre-miRs and anti-miRs (Figure 1b and Figure 2), pre-miRs (25 nM; Ambion,Thermo Fisher Scientific, Merelbeke, Belgium) and anti-miRs (25 nM; Ambion) were transfected into ECs and SMCs using Dharmafect-4 (Dharmacon) according to manufacturer’s instructions. Transfected ECs and SMCs were plated in EGM-2 or SMCM, respectively. After a 24-h transfection, cells were washed and kept in their media for an additional period, depending on the assay. For functional assays using EVs enriched in miR-539 and miR-582, ECs and SMCs were transfected with pre-miR-539 and pre-miR-582, respectively, as described above. After a 24 h transfection, cells were washed and kept for 72 h; EVs were isolated as described below and are referred to here as miR-539 EVs and miR-582 EVs.

### 4.9. Transwell Coculture Assay

For cocultures presented in Figure 1b, donor cells were seeded onto 6-well plates and incubated overnight. Then, 0.4-µm transwells (Corning) were added and recipient cells transfected with pre-miR-mmu-miR-298 (25 nM, Ambion) as described above and seeded onto the inner part of the transwell membranes. After 48 h of coculture, cells from the bottom chamber were collected and analyzed.

### 4.10. RNAse Treatment of EVs

Purified EV (50 µg) were subjected to 50 μg/mL RNAse A (#EN0531, Thermo Fisher Scientific), in a final volume of 50 μL, for 30 min at 37 °C. As control, EV preparations were pre-treated with SDS 1%, triton X-100, 0.1% for 10 min at RT. Preparations were then resuspended in qiazol and purified as described in Section 4.11.

### 4.11. Coculture and microRNA Profiling of EVs

For the experiments described in Section 2.2, EVs were produced by ECs and SMCs cultured as a monoculture or in coculture. For coculture experiments, 1,200,000 ECs were seeded in T-75 flasks in EGM-2 medium supplemented with 5% EV-depleted serum (half-confluency). After adhesion of the ECs for 1 h, 1,200,000 SCMs were added and cultured for 72 h. Cells were trypsinized and then separated with CD31 MicroBeads (Miltenyi Biotec, Bergisch Gladbach, Germany) in accordance with the manufacturer’s instructions. In brief, for 10 × 10^6^ cells, cells were resuspended in 60 µL of medium. Cells were incubated with 20µL FcR blocking Reagent and 20 μL de CD31 MicroBeads for 15 min at 4 °C. A total of 1 mL of medium is added and the mix is loaded on an MS column mounted on a MIDIMACS separator. SCMs were recovered in the flow-through. The column was washed three times with medium. ECs were eluted in 3 mL of medium. Separated cells were centrifuged at 400× *g* for 5 min and seeded in in new flasks in EGM-2 medium supplemented with 5% EV-depleted serum. EVs were collected after 72 h and purified as described above. In parallel, for comparison, EVs were isolated from ECs and SMCs cultured for 72 h in EGM-2 medium supplemented with 5% EV-depleted serum. Total RNA of 100 µg of EVs was extracted with the miRNeasy mini kit (Qiagen Thermo Fisher Scientific) following the manufacturer protocol. Reverse transcription was performed using the miRCURY LNA Universal RT microRNA PCR kit (Exiqon, Thermo Fisher Scientific). cDNAs were then mixed with ExiLENT SYBR Green master mix (Exiqon) and the ROX Passive Reference Dye (Bio-Rad, Temse, Belgium) according to the manufacturer’s instructions. Quantitative PCR was performed following manufacturer’s instructions on a microRNA Ready-to-Use PCR panel 1 V3.M (Exiqon). The controls included reference genes, inter-plate calibrators, and negative controls.

### 4.12. miRNA Expression Analysis

For RNA isolation from cells or EVs (experiments in Section 2.1, Section 2.2, Section 2.3 and Section 2.4), total RNA was extracted with the miRNeasy mini kit (Qiagen) following the manufacturer’s protocol. For EV samples, 6.25 fmol of Cel-miR-39 and cel-miR-238 were spiked into 5 µg of purified EVs before performing the RNA extraction. TaqMan assays were used to assess miRNA expression; 10 ng RNA were reverse-transcribed into cDNA using the TaqMan microRNA Reverse Transcription kit and the TaqMan microRNA assay primers (Applied Biosystems, Foster City, CA).). qPCR was performed with the resulting cDNA using the TaqMan microRNA primers and TaqMan Universal PCR master mix reagents (Applied Biosystems). Thermal cycling was performed on an Applied Biosystems 7900 HT detection system (Applied Biosystems). For cells, the relative miRNA levels were normalized to RNU44 and RNU48, two internal controls. For EVs, the relative miRNA levels were normalized to cel-miR-39 and cel-miR-238, two spiked-in miRNAs.

### 4.13. Proliferation Assays

Cell proliferation was measured following the BrdU incorporation. For manipulations using cells transfected with pre-miR and anti-miRs (Figure 2), ECs were transfected and seeded onto 96-well plates for 66 h. BrdU was added for 8 h, and proliferation was analyzed using the Cell Proliferation ELISA BrdU (Colorimetric) kit (Roche) following the manufacturer’s instructions. SMCs were transfected and seeded onto 96-well plates for 48 h. BrdU was added for 24 h, and proliferation was then analyzed. For manipulations using EVs (Figure 3), cells were seeded and incubated in an EVs-depleted medium. The day after, cells were treated with 15 µg/mL of EVs for 48 h. BrdU was added for the last 8 h (EC) or 24 h (SMC).

### 4.14. Migration Assay for SMCs in Boyden Chambers

For manipulations using cells transfected with pre-miR and anti-miRs (Figure 2), transfected SMCs were seeded into 8 µm, 24-well Boyden chambers (Costar). The lower chamber was filled with 750 µL of SMCM supplemented with 20 ng/mL PDGF-BB (Reliatech, WolfenbüttelGermany). The cells were placed in 300 µL of b-SMCM (ScienCell) in the upper chamber and allowed to migrate through the membrane for 6 h at 37 °C. After fixation with cold methanol, cells were stained with 4% Giemsa and counted on the lower side of the membrane using ImageJ software. For experiments using EVs (Figure 3), 15 µg/mL EVs were added to the SMCs in the upper chamber before the 6 h migration.

### 4.15. Migration Healing Assay of ECs

For manipulations using cells transfected with pre-miR and anti-miRs (Figure 2), transfected ECs were plated into 48-well plates and cultured in EGM-2. To examine migration, the confluent monolayers were mechanically scratched using a pipette tip to create a wound. Cells were washed with PBS and EBM-2 0.5% DBS was added to allow for migration. The distance between the two sides of the wound was measured with a graduated ocular lens coupled with an Olympus CKX41 microscope (Olympus). The distance between the two sides of the wound after 6 h of migration was subtracted from the distance at Time 0 and represented on a graph. For experiments using EVs (Figure 3), cells were incubated with 15 µg/mL EVs for 24 h before the scratch.

### 4.16. Tubulogenesis Assay

For manipulations using cells transfected with pre-miR and anti-miRs (Figure 2), transfected ECs were seeded onto a matrigel-coated 96-well plate and allowed to form tubes during 16 h at 37 °C. Pictures were taken using an Olympus CKX41 microscope (Olympus) and the number of branching tubes was analyzed using ImageJ software and its Angiogenesis Analyzer plugin (http://imagej.nih.gov/ij/macros/toolsets/Angiogenesis%20Analyzer.txt, accessed on 6 April 2019). For experiments using EVs (Figure 3), cells were seeded and directly incubated with 15 µg/mL EVs for 16 h to allow for the formation of tubes.

### 4.17. Next-Generation Sequencing

To analyze the genes regulated by miR-539 and miR-582 in experiments described in Section 2.5, total RNA was extracted with the miRNeasy mini kit (Qiagen) following the manufacturer’s protocol. RNA quality was measured with the RNA 6000 Nano kit (Agilent) on a 2100 Bioanalyzer (Agilent). cDNA libraries were generated from 1 µg of RNA and the TruSeq Stranded Total RNA Sample Preparation with Ribo-Zero kit (Illumina) according to manufacturer’s instructions. Libraries were indexed then sequenced with a NextSeq500 sequencer (Illumina). Data were analyzed with the TopHat and Cufflinks Assembly & Differential Expression tools available on BaseSpace (http://basespace.illumina.com, accessed on 25 November 2015). The results were compared with the data available on TargetScan version 7.0 (http://www.targetscan.org, accessed on 3 February 2016), and the biological processes were highlighted thanks to the Gene Ontology database via the STRING web tool (https://string-db.org, accessed on 23 February 2016). Heatmap were generated by Clustvis web tool [58].

### 4.18. mRNA Expression Analysis

mRNA expression analysis (experiment in Section 2.5) was performed by qPCR as follow: total RNA was extracted from cells with the miRNeasy mini kit (Qiagen) following the manufacturer’s protocol. cDNA synthesis was performed with 500 ng of total RNA and the iScript cDNA Synthesis kit (BioRad), according to the manufacturer’s instructions. A total of 20 ng of cDNA was mixed with the Takyon MasterMix (Eurogentec, Seraing Belgium). Thermal cycling was performed on an Applied Biosystems 7900 HT detection system (Applied Biosystems). For all reactions, random RNA preparations were also subjected to sham reverse transcription to verify the absence of genomic DNA amplification. The relative transcript level of each gene was normalized to the housekeeping genes cyclophilin-A (PPIA) and glyceraldehyde 3-phosphate dehydrogenase (GAPDH). Primers used for the qPCR are listed in Table 2.

### 4.19. Sequences of qPCR Primers

**Table 2:** Sequences of the primers used for the qPCR. All primers were synthesized by IDT-DNA. ADM: adrénomédulline, AKT1: AKT serine/threonine kinase 1, CASP3: caspase 3, CAV2: cavéoline 2, CDK6: *Cyclin-Dependent Kinase 6*, CXCL1: *C-X-C Motif Chemokine Ligand 1*, FGF2: *Fibroblast Growth Factor 2*, ITGA3: *integrin subunit alpha 3*, ITGB3: *integrin subunit beta 3*, MAP3K7: *Mitogen-Activated Protein Kinase Kinase Kinase 7*, PDCD6: *Programmed Cell Death 6*, PHB2: prohibitine 2, PTPRJ: *Protein Tyrosine Phosphatase Receptor Type J*, ROBO2: *Roundabout Guidance Receptor 2*, TP53INP1: *Tumor Protein P53 Inducible Nuclear Protein 1*, VASH1: *Vasohibin 1*, VCAM-1: *Vascular Cell Adhesion Molecule-1*.

Name	Forward Primer	Reverse Primer
ADM	ATGAAGCTGGTTTCCGTCG	GACATCCGCAGTTCCCTCTT
AKT1	AGCGACGTGGCTATTGTGAAG	GCCATCATTCTTGAGGAGGAAGT
CASP3	CATGGAAGCGAATCAATGGACT	CTGTACCAGACCGAGATGTCA
CAV2	AAGACCTGCCTAATGGTTCTGC	CTCGTACACAATGGAGCAATGAT
CDK6	TGCCCGCATCTATAGTTTCCA	AGCCAACACTCCAGAGATCCAC
CXCL1	CTTGCCTCAATCCTGCATC	CCTTCTGGTCAGTTGGATTTG
FGF2	CACATTTAGAAGCCAGTAATCT	CCCGACGGCCGAG TTGAC
ITGA3	TGTGGCTTGGAGTGACTGTG	TCATTGCCTCGCACGTAGC
ITGB3	AGTAACCTGCGGATTGGCTTC	GTCACCTGGTCAGTTAGCGT
MAP3K7	ATTGTAGAGCTTCGGCAGTTATC	CTGTAAACACCAACTCATTGCG
PDCD6	ATGGCCGCCTACTCTTACC	TCCTGTCTTTATCGACCCTCTG
PHB2	GTGCGCGAATCTGTGTTCAC	GATAATGGGGTACTGGAACCAAG
PTPRJ	GGCACCCCTAGTCCAATTCC	TCCCATTAGATCCTTGTTCAGGT
ROBO2	GTTTGTGTTGCGAGGAACTATCT	GTTTTGTCGGAAGTCATCTCGTA
TP53INP1	TTCCTCCAACCAAGAACCAGA	GCTCAGTAGGTGACTCTTCACT
VASH1	GGTGGGCTACCTGTGGATG	CACTCGGTATGGGGATCTTGG
VCAM-1	GAGACCACCCCAGAATCTAGATA	ACTTCCTGTCTGCATCCTCCA

### 4.20. Retinal Murine Neovascularization Model

C57BL/6 mice were used in this study and purchased from Elevage Janvier (France). Mice were maintained and handled according to the protocol approved by the Institutional Ethics Committee of the University of Liège (Protocol #1387). EVs injected in mice have been produced from human cells as described in Section 4.2. and loaded with pre-miRs as described in Section 4.8. To analyze postnatal neovascularization in mouse retina, pups were intra-peritoneally (IP) injected at Postnatal Day 1 (P1) with 20 µg of miR-539 EVs and sacrificed at P7. Their eyes were fixed in 4% paraformaldehyde in PBS for 45 min, and their retinas were dissected, incubated with biotinylated Isolectin B4 (Vector laboratories), and stained with streptavidin (Alexa 488 Invitrogen) and CY3-anti-α smooth muscle actin before being flat-mounted, as previously described [59]. Vascularization was assessed by measuring the radial expansion, the numbers of branches, and the total length of vessels. The radial expansion was calculated as the ratio between the vascular radius and the retinal radius. The retinal radius (from the optic nerve to the edge of the retina) and the vascular radius (from the optic nerve to the vascular front) of each petal of the retina were measured. The value for each retina was calculated as the mean of the radii for all petals. The number of branches and the total length of vessels were analyzed using ImageJ software and its Angiogenesis Analyzer plugin (http://imagej.nih.gov/ij/macros/toolsets/Angiogenesis%20Analyzer.txt, accessed on 30 July 2019). To analyze the impact of miR-582 EVs on postnatnal neovascularization and SMC/EC coverage, pups were injected at P7 with miR-582 EVs and scarified on P12. Neovascularization is assessed as described above on both the superficial vascular plexus in Layer 1 (L1) and in the deeper vascular plexus in Layer 3 (L3) [59]. The smooth muscle/endothelial cell coverage was calculated using the coloc2 plugin (https://imagej.net/Coloc_2, accessed on 30 July 2019) on isolectin-B4/α-SMA stained slides. From five to nine pups were used in each group. Pictures were taken using a confocal Zeiss LSM700 microscope (equipped with 10X/0.25, 20X/0.50NA, 40X/1.3NA and 63X/1.4NA objectives).

### 4.21. Statistical Analysis

All in vitro experiments were performed in triplicate and were repeated at least three times unless otherwise indicated. The in vitro proliferation, migration, and tubulogenesis assays were calculated relative to the controls in each experiment, and general linear model univariate analysis was used to determine differences between treatment groups, using experiment as covariate. All other data were analyzed with the Student’s *t*-test or one-way ANOVA for multiple comparison. All analyses were performed using Prism software (GraphPad Software), and *p* values less than 0.05 were considered statistically significant.

## Figures and Tables

**Table 1:** miR-539 and miR-582 target many genes in ECs and SMCs. Genes regulated by miR-539 and miR-582 in ECs and SMCs according to the data obtained by RNA sequencing. Data were compared with the lists of predicted target genes available on TargetScan and then analyzed with Gene Ontology to highlight the biological processes where identified target genes are involved.

GO ID	Biological Processes	Matched Genes in the Network	Genes in the Networks	False Discovery Rate
miR-539 in ECs				
GO:0030336	negative regulation of cell migration	28	237	0.00093
GO:0008284	positive regulation of cell population proliferation	69	878	0.002
miR-582 in ECs				
GO:0035295	tube development	59	793	1.37 × 10^5^
GO:0008284	positive regulation of cell population proliferation	58	878	0.00035
GO:0001568	blood vessel development	44	464	1.49 × 10^6^
GO:0001525	angiogenesis	30	297	4.72 × 10^5^
miR-539 in SMCs				
GO:0008285	negative regulation of cell population proliferation	18	669	0.0247
GO:0030334	regulation of cell migration	20	753	0.0194
GO:0045765	regulation of angiogenesis	14	277	0.0044
miR-582 in SMCs				
GO:0008285	negative regulation of cell population proliferation	103	669	0.0016
GO:0030335	positive regulation of cell migration	81	452	0.00015
GO:1901342	regulation of vasculature development	58	305	0.00067

## Data Availability

The data that support the findings of this study are available upon reasonable request to the corresponding author.

## Supplementary Materials

The following supporting information can be downloaded at: https://www.mdpi.com/article/10.3390/ijms23010331/s1.
