# Supplementary material for: Extracellular Vesicles Mediate Communication between Endothelial and Vascular Smooth Muscle Cells
Source: Int J Mol Sci. 2021 Dec 28;23(1):331. doi: 10.3390/ijms23010331 (PMC8745747; doi:10.3390/ijms23010331)
Supplement: Supplementary file 1 [file ijms-23-00331-s001.zip › ijms-1480954-supplementary/Supplementary figures_fontaine_revision.pdf]

Supplementary material for

Extracellular vesicles mediated communication between endothelial and vascular smooth muscle cells.

Marie Fontaine[1], Stéphanie Herkenne[1], Olivier Ek[1], Alicia Paquot[1], Amandine Boeckx[1], Cécile Paques[1], Olivier Nivelles[1], Marc Thiry[2] and Ingrid Struman[1]

[1] *Laboratory of Molecular Angiogenesis, GIGA Research center, University of Liège, Belgium*

[2] *Laboratory of Cell and Tissues Biology, University of Liège, Belgium*

Corresponding author:

Ingrid Struman, PhD

Address: *Laboratory of Molecular Angiogenesis, University of Liège, GIGA Research Center, B34, 1 avenue de l'hôpital, 4000 Sart Tilman, Belgium*

Phone: +32-43663566

Email: i.struman@uliege.be

ORCID : 0000-0003-2550-3707

Supplementary Figures

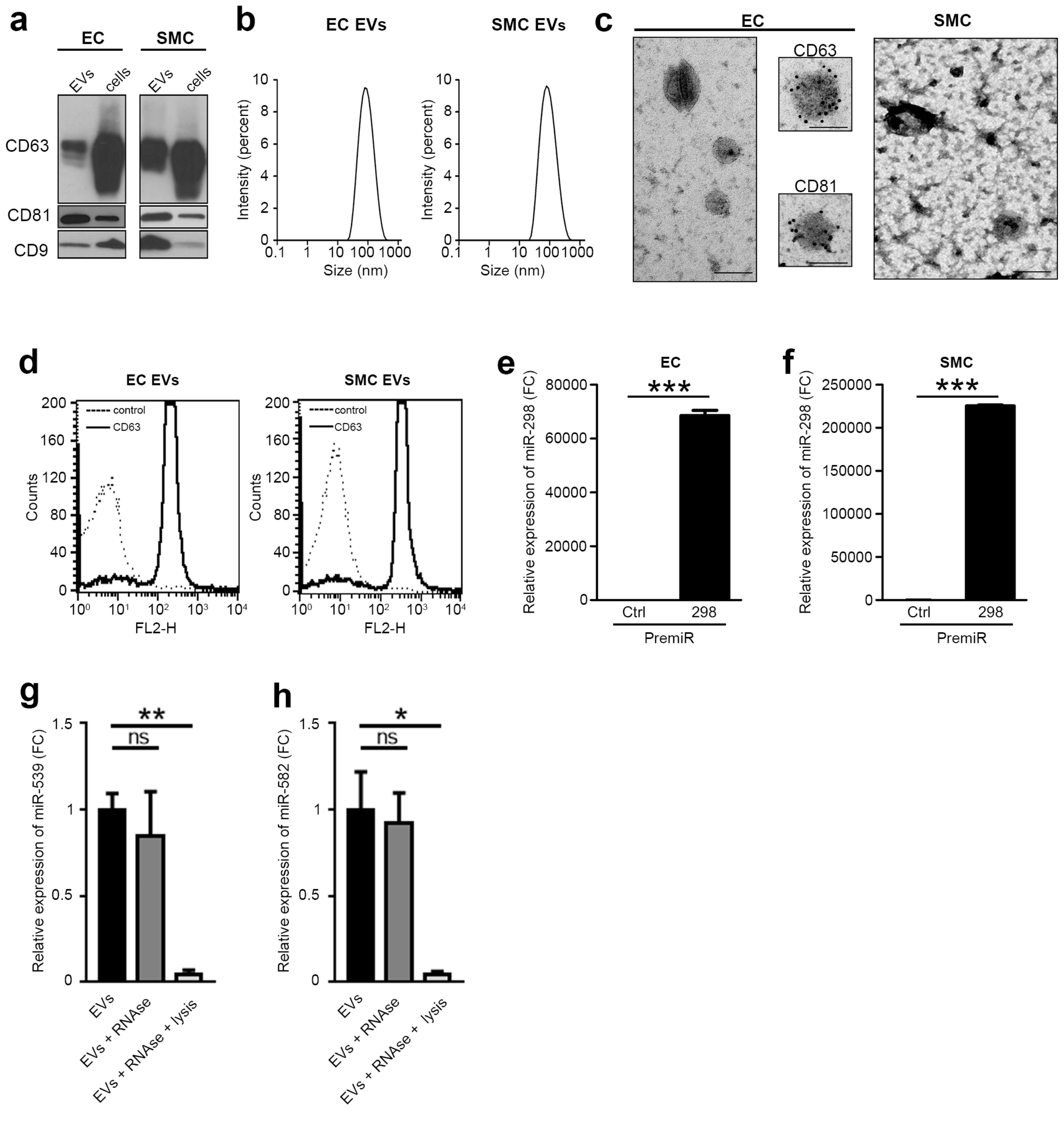

Figure S1: Extracellular vesicles characterization

EVs were purified from ECs and SMCs by ultracentrifugation and characterized as follow (a-d): (a) Western blotting of CD63, CD81 and CD9 in EC EVs, SMC EVs and cells, (b) Dynamic light scattering analysis of EC EVs (max = 90 nm) and SMC EVs (max = 80 nm), (c) Electron micrographs of ECs and SMCs EVs labeled with CD63 and CD81, scale bars= 100 nm, (d) Flow cytometry analysis of EC EVs and SMC EVs immunolabeled for CD63. (e) miR-298 levels evaluated using qPCR in ECs transfected with pre-miR-control or pre-miR-298. (f) miR-298 levels evaluated using qPCR in SMCs transfected with pre-miR-control or pre-miR-298. (g, h) Treatment of purified EVs by RNAse. 50 µg of EVs purified from ECs (g) and SMCs (h) were treated with or without RNase A and/or lysis buffer containing Triton X-100 and SDS before RNA extraction. All data are the mean ± SD (n ≥ 3). **P*<0.05, ***P*=0.01, *** P < 0.001 *vs.* the respective control.

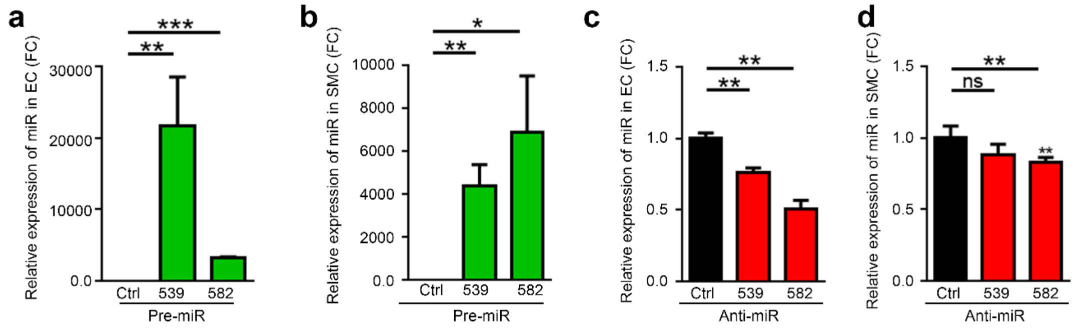

Figure S2: Transfections efficiency with Pre-miR and Anti-miR

(a) microRNA levels evaluated using qPCR in ECs transfected with pre-miR-control or pre-miR-539 or pre-miR-582. (b) microRNA levels evaluated using qPCR in SMCs transfected with pre-miR-control or pre-miR-539 or pre-miR-582. (c) microRNA levels evaluated using qPCR in ECs transfected with anti-miR-control or anti-miR-539 or anti-miR-582. (d) microRNA levels evaluated using qPCR in SMCs transfected with anti-miR-control or anti-miR-539 or anti-miR-582. All data are the mean ± SD ($n \geq 3$). * $P < 0.05$, ** $P < 0.01$ and *** $P < 0.001$ *vs.* the respective control.

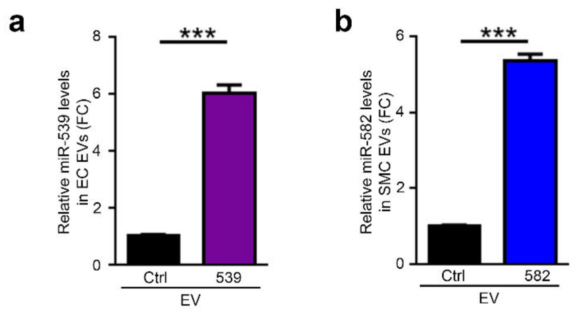

Figure S3. miR levels in EVs from cells transfected with the Pre-miRs

(a) MiR-539 levels evaluated using qRT-PCR in ECs EVs produced by ECs transfected with pre-miR-control (Ctrl EV) or pre-miR-539 (539 EV). (b) miR-582 levels evaluated using qRT-PCR in SMCs EVs produced by SMCs transfected with pre-miR-control (Ctrl EV) or pre-miR-582 (582 EV). All data are the mean ± SD ($n \geq 3$). *** $P < 0.001$ *vs.* the respective control.

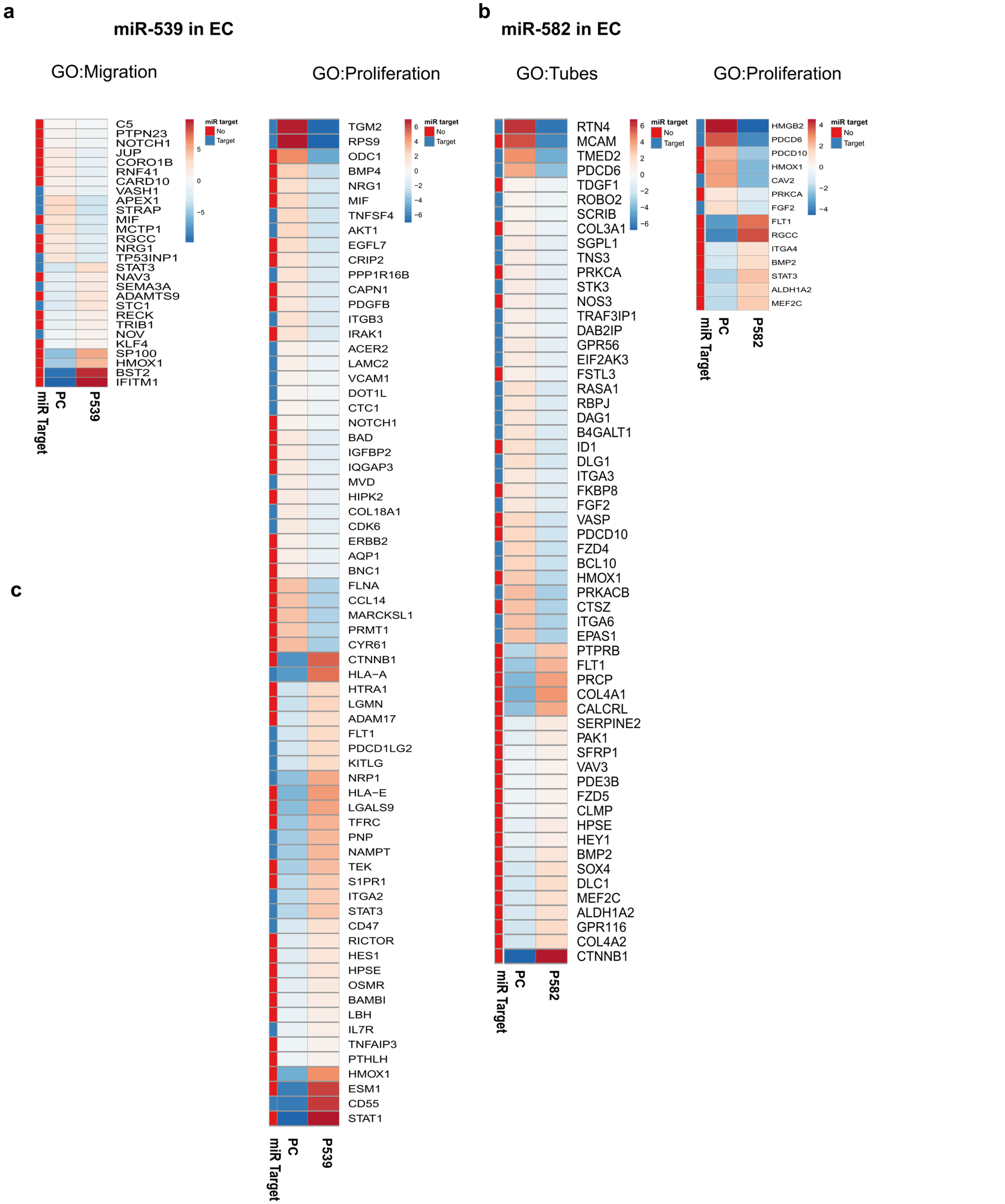

Figure S4

miR-539 and miR-582 regulate genes involved in angiogenesis-related biological processes ECs. (a-b) Heatmap of genes lists taken from gene ontology (GO) networks. RNA sequencing analyses were performed on ECs transfected with pre-miR-539 (a), pre-miR-582 (b). Significantly regulated genes were subjected to gene ontology analysis. From each list, potential gene targets for the microRNA were identified by TargetScan analysis. GO networks involved in regulation of angiogenesis are shown.

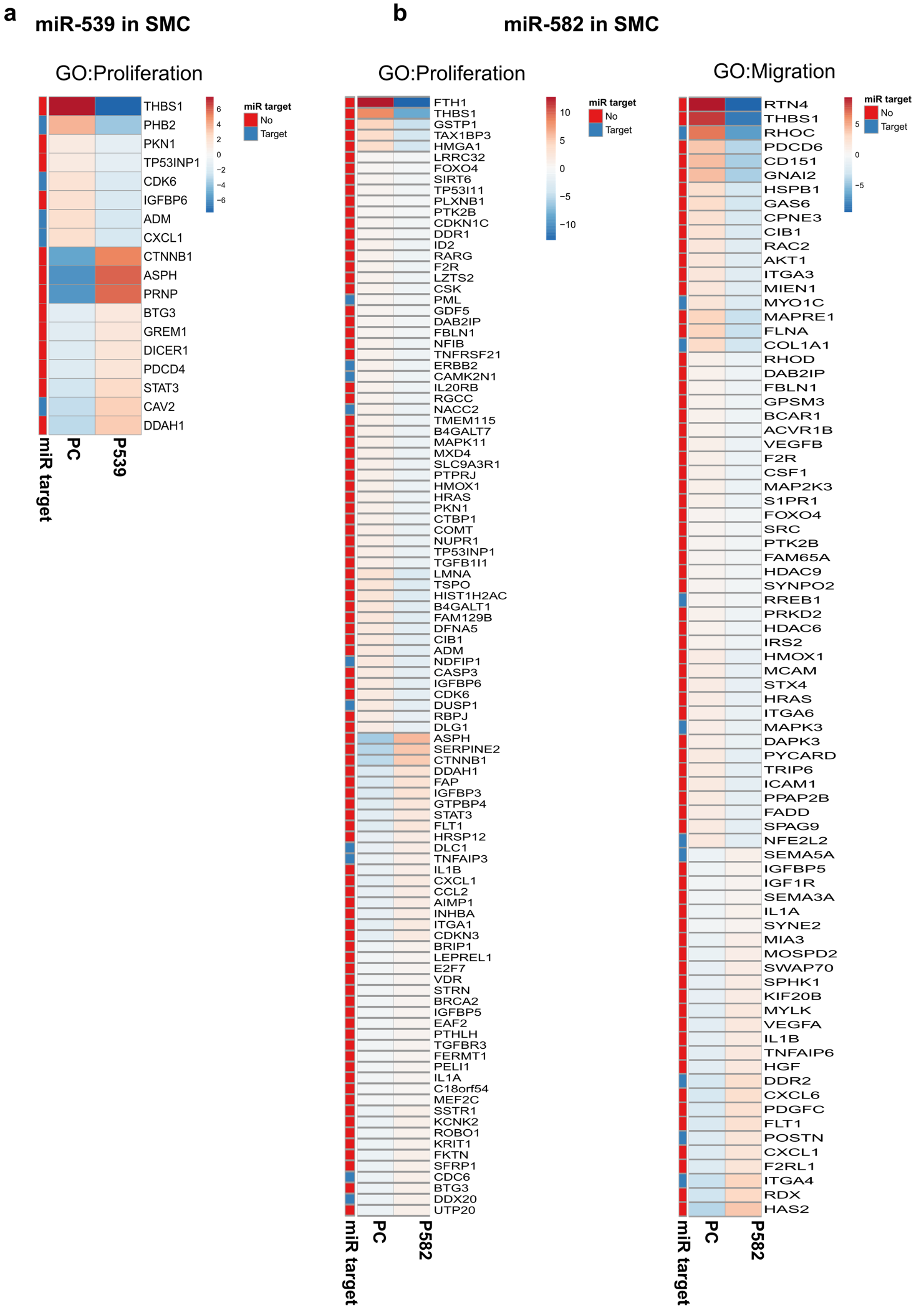

Figure S5

miR-582 regulate genes involved in angiogenesis-related biological processes ECs and SMCs. (a-b) Heatmap of genes lists taken from gene ontology (GO) networks. RNA sequencing analyses were performed VSMCs transfected with pre-miR-539 (a) and pre-miR-582 (b). Significantly regulated genes were subjected to gene ontology analysis. From each list, potential gene targets for the microRNA were identified by TargetScan analysis. GO networks involved in regulation of angiogenesis are shown.

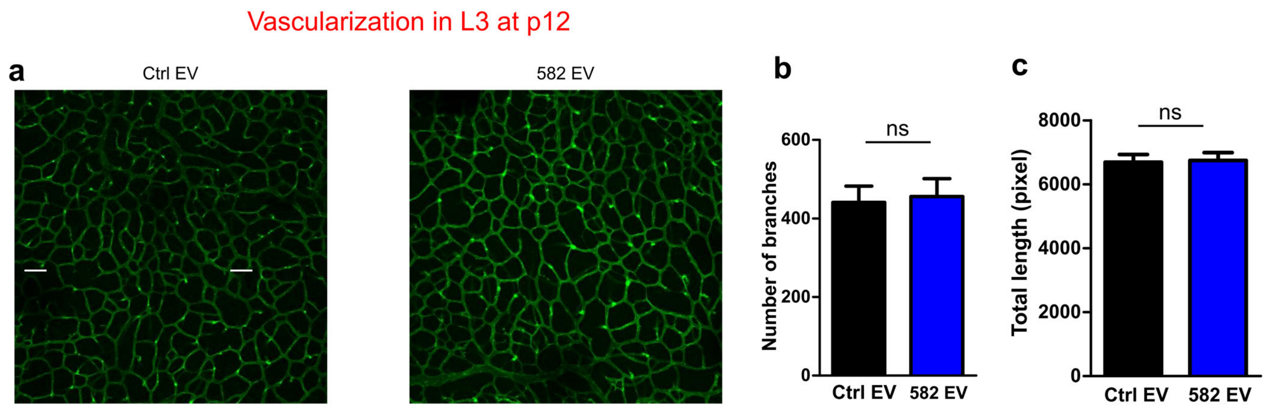

Figure S6

miR-582 EVs does not affect neovascularization in the L3 (a). EVs were purified from SMCs transfected with pre-miR-582 (582 EV) on a pre-miR-control (Ctrl EV) (a) Confocal images of isolectin-B4 staining in the Deeper layer (L3) on postnatal day 12 retinas from pups that were injected at postnatal day 7 with EVs. Scale bar: 200 µm. The histograms represent (b), the number of branches (c) and the total length of isolectin-B4+ vessels, N=8 eyes, 2 independent experiments.

<u>Supplementary Table</u>

Supplementary table 1

microRNA qPCR profiling on EVs produced by ECs alone, SMCs alone or cells that were in coculture. List of microRNAs that are differentially present in EVs isolated from ECs and SMCs before and after co-culture.

Supplementary table 2

RNA sequencing on total RNAs from ECs and SMCs transfected with microRNA mimics. List of genes that are differentially expressed in ECs and SMCs transfected with miR-539 or miR-582 mimics.
